# Characterization of the antibacterial and biofilm inhibitory activities of *Arctium lappa* L. (greater burdock) against representative members of the ESKAPE group

**DOI:** 10.3389/fphar.2026.1941242

**Published:** 2026-09-16

**Authors:** Danae Kala Rodriguez Bardaji, Gabriella Fedus, Samantha Tran, Dinindu de Silva, Lily Fagan, Michael A. Savka, Dawn Carter, Nagela Bernadelli Sousa Silva, André O. Hudson

**Affiliations:** 1 Thomas H. Gosnell School of Life Sciences, College of Science, Rochester Institute of Technology, Rochester, NY, United States; 2 Department of Biomedical Engineering, Rochester Institute of Technology, Rochester, NY, United States; 3 Laboratory of Antimicrobial Testing, Institute of Biomedical Sciences (ICBIM), Federal University of Uberlândia, Uberlândia, Minas Gerais, Brazil

**Keywords:** antibacterial activity, antibiotics, antimicrobial resistance, Arctium lappa, biofilm inhibition, biosynthetic gene clusters, ESKAPE, genome mining

## Abstract

Antimicrobial resistance creates an urgent need for new antibacterial strategies, and medicinal plants remain an important source of chemically diverse bioactive metabolites. This study investigated the antibacterial potential of *Arctium lappa* L. (greater burdock) naturalized in the United States using a genome-guided approach integrated with microbiological and mechanistic assays. Genome mining identified 35 putative biosynthetic gene clusters associated with diverse specialized metabolite classes. An 80% aqueous ethanol leaf extract was evaluated against six ESKAPE-like bacterial strains: *Enterococcus faecium* RIT857 and *Staphylococcus aureus* ATCC 25923 (Gram-positive), and *Klebsiella aerogenes* RIT3131, *Acinetobacter* sp. RIT587, *Pseudomonas aeruginosa* ATCC 27853, and *Enterobacter* sp. RIT637 (Gram-negative). MIC and MBC values ranged from 64 to 512 and 128–512 μg/mL, respectively, while MBIC_50_ values ranged from 64 to 256 μg/mL. Time-kill assays demonstrated bactericidal activity, with Gram-negative strains reaching a ≥3-log10 reduction within 6 h and Gram-positive strains showing slower killing kinetics. LIVE/DEAD staining, scanning electron microscopy, and atomic force microscopy showed changes consistent with compromised membrane integrity, while H2DCFDA assays showed increased intracellular ROS in *S. aureus* (181% ± 11%) and *P. aeruginosa* (214% ± 13%) relative to untreated controls. A modified disk diffusion assay also showed increased inhibition zones for selected extract–antibiotic combinations. The individual metabolites responsible for these effects were not chemically identified, which limits direct linkage between predicted biosynthetic pathways and observed activity. Overall, the findings demonstrate antibacterial and biofilm-inhibitory activity of *A. lappa* extract and support further bioassay-guided fractionation and metabolomic characterization.

## 1 Introduction

Antimicrobial resistance (AMR) is one of the greatest challenges facing modern medicine, threatening the effective treatment of bacterial infections and increasing morbidity, mortality, and healthcare costs worldwide (Antimicrobial Resistance Collaborators, 2022; Centers for Disease Control and Prevention, 2019; World Health Organization, 2024a). Among the pathogens of greatest concern are the ESKAPE group, which includes *Enterococcus faecium*, *Staphylococcus aureus*, *Klebsiella aerogenes*, *Acinetobacter* spp., *Pseudomonas aeruginosa*, and *Enterobacter* spp. These organisms are responsible for a large proportion of healthcare-associated infections and possess diverse mechanisms of resistance, including reduced membrane permeability, active efflux systems, enzymatic antibiotic inactivation, and biofilm formation (Pendleton et al., 2013; Rice, 2008; Ciofu and Tolker-Nielsen, 2019). The continued emergence of multidrug-resistant strains, together with the limited development of new antibacterial agents, underscores the urgent need for alternative strategies for antibiotic discovery (Antimicrobial Resistance Collaborators, 2022; World Health Organization, 2024b).

Natural products have historically provided the structural foundation for many clinically important antibiotics, and they continue to represent one of the richest sources of chemically diverse bioactive molecules (Newman and Cragg, 2020; Atanasov et al., 2021). Although microorganisms have been the primary focus of natural product discovery efforts, medicinal plants synthesize a wide variety of specialized metabolites that contribute to defense against environmental stressors and microbial pathogens (Atanasov et al., 2021; Dias et al., 2012). However, despite their long history of use in traditional medicine, many medicinal plants remain poorly characterized at the genomic level, limiting our understanding of their biosynthetic potential and their value as sources of novel antibacterial metabolites.

*Arctium lappa* L. (greater burdock), a member of the Asteraceae family, has been widely used in traditional medicine and has been reported to contain lignans, terpenoids, phenolic acids, and other classes of secondary metabolites with diverse biological activities (Navarro-Muñoz et al., 2020; Bardaji et al., 2025b). Nevertheless, the genomic basis underlying the biosynthesis of these metabolites and their potential contribution to antibacterial activity remain largely unexplored. Recent advances in whole-genome sequencing and biosynthetic gene cluster prediction provide an opportunity to integrate genomic information with functional studies to better understand the antimicrobial potential of medicinal plants (Bahramian et al., 2023; Perez-Delgado et al., 2021).

The objective of this study was to investigate the antibacterial potential of *A. lappa* naturalized in the United States using a genome-guided approach. By integrating genomic analysis with microbiological and mechanistic characterization, this work aims to provide new insights into the biosynthetic capacity and antibacterial properties of this medicinal species and to establish a framework for future natural product discovery.

## 2 Materials and methods

### 2.1 Plant material preparation

Leaves of *A. lappa* L. (Asteraceae) were collected in Rochester, New York, United States on the campus of the Rochester Institute of Technology (43.0847°N latitude and 77.6761°W longitude) Species identity was confirmed by genomic sequencing of both nuclear and chloroplast genomes (Bardaji et al., 2025a, Bardaji et al., 2026). In addition, morphological characteristics of the collected specimens, including features of the leaves and fruits, were consistent with those described for *A. lappa* (Figure 1), providing independent phenotypic support for the molecular identification. No voucher specimen was deposited; however, species identity was independently assessed through both genomic and morphological evidence. The accepted botanical name and taxonomic status of *A. lappa* L. were verified using the Medicinal Plant Names Services (MPNS) of the Royal Botanic Gardens, Kew, in accordance with recommendations for reporting plant materials.

**FIGURE 1:**
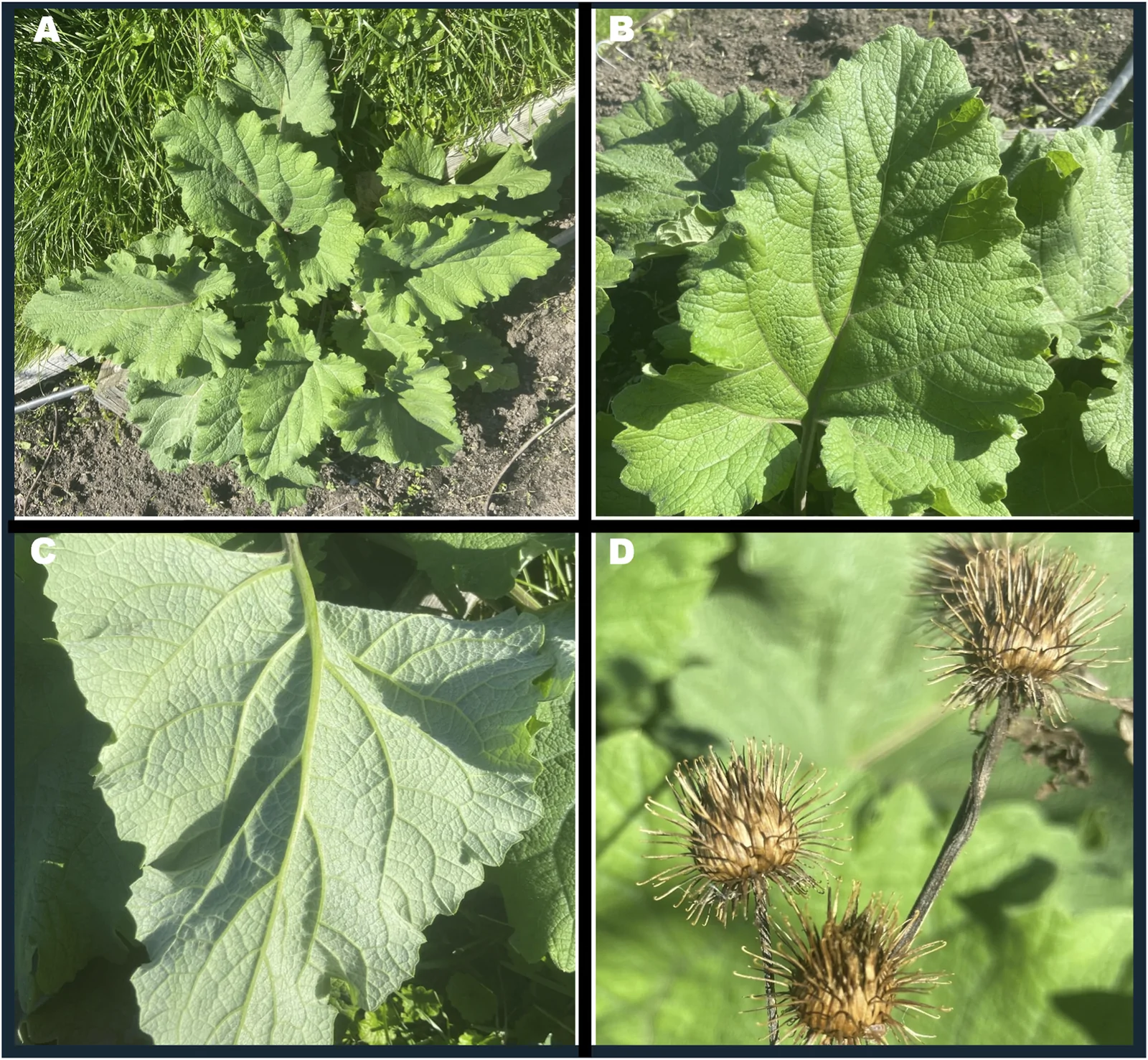
*Arctium lappa* L. (greater burdock) used in this study. **(A)** Representative whole plant showing the characteristic large, broad leaves. **(B)** Close-up of the upper leaf surface showing its characteristic morphology and prominent venation. **(C)** Abaxial (lower) leaf surface showing the dense pubescence and prominent venation. **(D)** Mature burrs showing the characteristic hooked involucral bracts associated with seed dispersal.

Leaves of *A. lappa* were selected for extraction based on their traditional medicinal use and phytochemical potential. Freshly harvested leaves were rinsed with tap water, freeze-dried using a Harvest Right Home Freeze Dryer (United States), and ground into a fine powder using an electric blender. The resulting homogeneous plant powder was subjected to solvent extraction.

Extraction was performed via maceration using solvents of varying polarity: methanol, ethanol, acetone, hexane, ethyl acetate, aqueous methanol (80:20 v/v), and aqueous ethanol (80:20 v/v). For each condition, the plant powder was mixed with solvent at a ratio of 1:10 (w/v) and incubated in an orbital shaker at 80 rpm for 48 h at 20 °C. The resulting mixtures were filtered through Whatman No. 1 filter paper to remove solid residues. The filtrates were concentrated using a rotary evaporator (BUCHI, United States) to obtain dried crude extracts, which were stored at 4 °C until further use. The extraction procedure was based on a conventional maceration method for medicinal plant materials (Abubakar and Haque, 2020). Initial antimicrobial screening by disk diffusion against ESKAPE-like bacterial strains (Section 2.3) revealed that the aqueous ethanol extract exhibited the most promising activity. Therefore, subsequent experiments were carried out exclusively with this extract. Extraction yields and preliminary screening results for all solvent extracts are provided in Supplementary Table S1.

### 2.2 Genome mining and biosynthetic gene cluster analysis

The genome of *A. lappa* was analyzed to investigate its biosynthetic potential using the draft genome sequence generated and described in our previously published dataset (Bardaji et al., 2025a). The genome sequencing data are publicly available in the National Center for Biotechnology Information (NCBI) under BioProject accession number PRJNA1167729 and BioSample accession number SAMN44012260. The draft genome was submitted to the PlantiSMASH v1.1 web server for automated identification and annotation of biosynthetic gene clusters (BGCs).

The analysis was performed using default parameters and focused on the detection of clusters containing three or more distinct biosynthetic enzyme subclasses, including terpene synthases (TPS), cytochrome P450 enzymes, type III polyketide synthases, methyltransferases, and glycosyltransferases. Each cluster was classified according to its predicted metabolic class (e.g., terpene, alkaloid, polyketide, flavonoid, or hybrid) and further analyzed based on the presence of core scaffold-forming enzymes and tailoring components.

Cluster architecture and gene content were visualized using PlantiSMASH’s interactive tools and manually curated to identify candidate BGCs with potential relevance to the antimicrobial activity of *A. lappa*.

### 2.3 Bacterial strains

Six ESKAPE-like bacterial strains were used to evaluate the antibacterial activity of the *A. lappa* extract. These included both reference strains and resistant clinical/environmental isolates from the RIT culture collection. These strains were selected for their phylogenetic similarity to the core ESKAPE group, their known antibiotic resistance profiles, and their relevance to nosocomial infections. The bacterial panel included *E. faecium* RIT857, *S. aureus* ATCC 25923, *K. aerogenes* RIT 3131, *Acinetobacter* sp. RIT 587, *P. aeruginosa* ATCC 27853, and *Enterobacter* sp. RIT637.

### 2.4 Determination of the minimum inhibitory concentration (MIC) and minimum bactericidal concentration (MBC)

The antibacterial activity of the 80% aqueous ethanol leaf extract of *A*. *lappa* was evaluated by determining the minimum inhibitory concentration (MIC) using the broth microdilution method according to the Clinical and Laboratory Standards Institute (CLSI) guidelines (CLSI, 2024). The extract was dissolved in dimethyl sulfoxide (DMSO) and serially diluted two-fold in Mueller–Hinton broth (MHB) in sterile 96-well microplates to obtain final concentrations ranging from 1 to 512 μg/mL. The final concentration of DMSO in the assay did not exceed 1% (v/v). Bacterial inoculum were prepared from overnight cultures and adjusted to approximately 5 × 10^5^ CFU/mL in each well.

Microplates were incubated at 37 °C for 24 h. Following incubation, 30 μL of 0.01% (w/v) aqueous resazurin solution was added to each well and the plates were incubated for an additional 30 min (Sarker et al., 2007). Wells that remained blue were considered indicative of growth inhibition, whereas wells that changed from blue to pink indicated metabolically active bacterial cells. Gentamicin was used as a positive control. Wells containing Mueller–Hinton broth only, bacterial inoculum without extract, and the highest concentration of DMSO used in the assay served as sterility, growth, and solvent controls, respectively. All experiments were performed in triplicate.

The minimum bactericidal concentration (MBC) was determined from wells showing no visible bacterial growth. Prior to the addition of resazurin, 10 μL aliquots from these wells were plated onto Mueller–Hinton agar and incubated at 37 °C for 24 h. The MBC was defined as the lowest concentration at which no bacterial colonies were observed. The antibacterial effect was classified as bactericidal when the MIC and MBC values were identical and bacteriostatic when the MBC exceeded the MIC. All assays were performed in triplicate. For interpretation of crude-extract antibacterial potency, MIC values < 100 μg/mL were considered significant, values > 100 to ≤625 μg/mL moderate, and values > 625 μg/mL weak, following the criteria summarized by Kuete (2010).

### 2.5 Time-kill kinetics assay

Time-kill assays were performed to evaluate the bactericidal activity of the 80% aqueous ethanol extract of *A*. *lappa* against ESKAPE-like bacterial strains following the general methodology described by Balouiri et al. (2016). Overnight cultures were adjusted to approximately 10^5^–10^6^ CFU/mL in MHB and incubated at 37 °C with shaking in the presence or absence of the extract. The extract was tested at the previously determined MBC for each strain. Untreated cultures served as growth controls, while gentamicin (for Gram-negative bacteria) or vancomycin (for Gram-positive bacteria), each tested at their respective MBCs, served as positive antibiotic controls.Aliquots were collected at 0, 2, 4, 6, 12, and 24 h, serially diluted in sterile phosphate-buffered saline (PBS), and plated onto Mueller–Hinton agar. Following incubation at 37 °C for 24 h, colonies were enumerated and expressed as log_10_ CFU/mL. Time-kill curves were generated by plotting viable bacterial counts against time. A reduction of ≥3 log_10_ CFU/mL relative to the initial inoculum was considered indicative of bactericidal activity. All experiments were performed in triplicate.

### 2.6 Biofilm inhibition assay

The biofilm inhibitory activity of the 80% aqueous ethanol extract of *A. lappa* was evaluated using a crystal violet biofilm quantification assay against six ESKAPE-like bacterial strains following O’Toole (2011) with modifications. Overnight cultures were diluted in MHB to an optical density at 600 nm (OD600) of 0.1. Aliquots were dispensed into sterile 96-well polystyrene microplates containing two-fold serial dilutions of the extract (1–512 μg/mL). Plates were incubated at 37 °C for 24 h under static conditions to allow biofilm formation.

Following incubation, planktonic cells were carefully removed, and wells were gently washed three times with sterile phosphate-buffered saline (PBS) to remove non-adherent bacteria. The attached biofilms were fixed, stained with 0.1% (w/v) crystal violet for 15 min, and washed thoroughly with distilled water to remove excess stain. The bound crystal violet was subsequently solubilized with ethanol, and the absorbance was measured at 600 nm using a microplate reader. Biofilm biomass was quantified as the absorbance of the solubilized crystal violet, with lower absorbance values indicating greater inhibition of biofilm formation. Untreated bacterial cultures served as the biofilm growth control. No separate reference antibiofilm compound was included in this assay; therefore, inhibition was quantified relative to the untreated control.

The minimum concentration required to inhibit 50% of biofilm biomass (MBIC_50_) was determined as the lowest extract concentration that reduced the measured biofilm biomass by approximately 50% relative to the untreated control. All experiments were performed in triplicate.

To investigate the mechanism of antibacterial activity, one representative Gram-positive (*S. aureus* ATCC 25923) and one representative Gram-negative (*P. aeruginosa* ATCC 27853) strain were selected from the initial screening panel. These strains were chosen because they displayed clear susceptibility to the *A. lappa* extract and are clinically relevant ESKAPE pathogens. Therefore, all subsequent mechanistic studies (SEM, AFM, LIVE/DEAD fluorescence staining, and ROS assays) were conducted using these representative organisms.

### 2.7 Scanning electron microscopy (SEM)

The effects of the 80% aqueous ethanol extract of *A*. *lappa* on bacterial cell morphology were evaluated by scanning electron microscopy (SEM) following the sample preparation protocol described by Parthasarathy (2019), with minor modifications. *Pseudomonas aeruginosa* ATCC 27853 and *Staphylococcus aureus* ATCC 25923 cultures were treated with the A. lappa extract at 4× MIC for 2 h. Untreated cultures served as controls. Following incubation, 10 μL of each culture was processed for SEM analysis. Untreated cultures served as morphological controls. A separate membrane-damaging positive control was not included because the SEM experiment was designed as a qualitative comparison between extract-treated and untreated cells.

Briefly, bacterial cells were fixed in 2% glutaraldehyde prepared in phosphate-buffered saline (PBS; pH 7.4) for 45 min at room temperature. Samples were washed three times for 5 min each with PBS and subsequently dehydrated through a graded ethanol series (50%, 60%, 70%, and 80%) for 10 min at each concentration, followed by two 5-min washes in 95% ethanol and three 15-min washes in fresh 100% ethanol. Residual ethanol was removed by pipetting, and samples were allowed to dry overnight at room temperature.

Prior to imaging, samples were sputter-coated with gold–palladium for 2 min using an SPI sputter coater. Micrographs were acquired at an accelerating voltage of 5 kV using a Vega3 Tescan field-emission scanning electron microscope housed in the Chemistry and Materials Science Facility at the Rochester Institute of Technology.

### 2.8 Atomic force microscopy (AFM) analysis

Atomic force microscopy (AFM) was used to evaluate morphological changes in *P. aeruginosa* ATCC 27853 and *S. aureus* ATCC 25923 following treatment with the 80% aqueous ethanol extract of *A. lappa*. Sample preparation was carried out according to the protocol described by Bolshakova et al. (2004), with minor modifications. Overnight cultures were diluted to approximately 1 × 10^7^ CFU/mL in Mueller–Hinton broth and exposed to the extract at their respective minimum bactericidal concentrations (MBCs) for 2 h at 37 °C under static conditions. Untreated cultures incubated under identical conditions served as controls. Untreated cultures served as morphological controls. A separate membrane-damaging positive control was not included in the AFM experiment.

Following treatment, bacterial cells were harvested by centrifugation (5,000 × g, 5 min), washed twice with sterile phosphate-buffered saline (PBS), and fixed with 2.5% glutaraldehyde in PBS for 1 h at room temperature. Fixed cells were deposited onto freshly cleaved mica discs and allowed to adhere for 30 min. Samples were gently rinsed with deionized water and air-dried overnight at room temperature.

AFM imaging was performed in tapping mode using a Multimode 8 atomic force microscope (Bruker, Billerica, MA, United States) equipped with silicon cantilevers (spring constant ∼40 N/m). Images were acquired under ambient conditions over scan areas of 5 × 5 μm and 20 × 20 μm at a scan rate of 1.0 Hz.

Cell morphology, surface topography, and membrane integrity were qualitatively compared between untreated and treated cells. Representative three-dimensional topographical images were acquired at scan areas of 5 × 5 μm and 20 × 20 μm. Structural alterations, including increased surface roughness, irregular surface topology, membrane deformation, and cell collapse, were considered indicative of extract-induced damage.

### 2.9 Cell viability and membrane integrity assessment by LIVE/DEAD fluorescence staining

The effects of the 80% aqueous ethanol extract of *A*. *lappa* on bacterial viability and membrane integrity were evaluated using the LIVE/DEAD™ BacLight™ Bacterial Viability Kit (Thermo Fisher Scientific, Waltham, MA, United States), following the methodology recommended by the manufacturer and the methodology described by Berney et al. (2007). *Staphylococcus aureus* ATCC 25923 and *P. aeruginosa* ATCC 27853 were grown in Mueller–Hinton broth to early exponential phase and adjusted to an OD600 of 0.2–0.3. Bacterial suspensions were exposed to the extract at 4× MIC for 2 h at 37 °C. A concentration of 4× MIC was selected for the mechanistic assays to ensure a rapid and reproducible antibacterial effect, thereby facilitating the detection of membrane damage and oxidative stress within the experimental incubation period. Final treatment concentrations were 512 μg/mL for *S. aureus* and 256 μg/mL for *P. aeruginosa*. Untreated and vehicle-treated cultures served as controls.

Following treatment, cells were collected by centrifugation, washed twice with sterile phosphate-buffered saline (PBS), and stained with SYTO 9 and propidium iodide (PI) according to the manufacturer’s instructions. Samples were incubated in the dark for 15 min at room temperature and transferred to 6-well plates for imaging.

Fluorescence images were acquired using an Olympus IX81 inverted fluorescence microscope equipped with a ×40 objective (Olympus Corporation, Tokyo, Japan) and appropriate filter sets for SYTO 9 and PI detection. SYTO 9 stains all bacterial cells, whereas PI penetrates only cells with compromised membranes. Cells exhibiting green fluorescence were considered viable, while cells exhibiting red fluorescence were considered membrane-damaged or nonviable. Representative images were acquired using identical microscope settings for untreated and treated samples to enable direct qualitative comparison. All experiments were performed in triplicate.

### 2.10 Reactive oxygen species (ROS) detection assay

Intracellular reactive oxygen species (ROS) production following treatment with the 80% aqueous ethanol extract of *A*. *lappa* was evaluated using 2′,7′-dichlorodihydrofluorescein diacetate (H_2_DCFDA; Thermo Fisher Scientific, Waltham, MA, United States) following the method described by Wang and Joseph (1999), with minor modifications. *Staphylococcus aureus* ATCC 25923 and *P. aeruginosa* ATCC 27853 were grown overnight in MHB, subcultured to the early exponential phase, and adjusted to an optical density at 600 nm (OD_600_) of 0.2–0.3.

A 10 mM H_2_DCFDA stock solution was prepared in anhydrous dimethyl sulfoxide (DMSO), aliquoted, and stored at −20 °C protected from light. Immediately before each experiment, a fresh 10 μM working solution was prepared in phosphate-buffered saline (PBS). Bacterial suspensions were incubated with H_2_DCFDA (10 μM) for 20 min at room temperature in the dark.

Following staining with H_2_DCFDA, 100 μL aliquots of the bacterial suspensions were transferred to sterile 96-well microplates and treated with the *A. lappa* extract at 4× MIC (512 μg/mL for *S. aureus* and 256 μg/mL for *P. aeruginosa*). Untreated cells and vehicle-control wells containing the same final concentration of DMSO (1%, v/v) as the extract-treated samples served as negative controls. Plates were incubated at 37 °C for 2 h. No exogenous ROS-inducing positive control was included; ROS changes were therefore interpreted relative to untreated and vehicle controls.

Fluorescence emission spectra were recorded using a Synergy H1 Hybrid Multi-Mode Microplate Reader (BioTek Instruments, Winooski, VT, United States) with an excitation wavelength of 485 nm and an emission scan from 500 to 600 nm. Fluorescence values at the emission maximum (535 nm) were normalized by dividing the relative fluorescence units (RFU) by the corresponding OD_600_ value for each well. The normalized fluorescence values were then expressed as a percentage relative to the untreated control, which was assigned a value of 100%. All experiments were performed in triplicate.

### 2.11 Antibiotic interaction assay using antibiotic disks and *Arctium lappa* extract

Potential interactions between the 80% aqueous ethanol extract of *A lappa* and selected antibiotics were evaluated using a modified disk diffusion assay based on the Kirby–Bauer method (Bauer et al., 1966), with minor modifications. Overnight bacterial cultures were adjusted to a 0.5 McFarland standard and incorporated into molten Mueller–Hinton agar at a ratio of 1:100 (200 μL bacterial suspension in 20 mL agar). The inoculated agar was poured into Petri dishes and allowed to solidify.

Commercial antibiotic disks were placed onto the agar surface under two experimental conditions: (1) antibiotic disk alone and (2) antibiotic disk supplemented with 10 μL of *A. lappa* extract (512 μg/mL) applied directly onto the disk. Plates were incubated at 37 °C for 24 h.

Zones of inhibition were measured in millimeters using ImageJ software following calibration with the 6-mm antibiotic disk diameter. Potential interactions were inferred when the combination of extract and antibiotic produced a larger inhibition zone than the corresponding antibiotic alone. Assays were performed in triplicate, and the mean zone diameter was used for analysis.

### 2.12 Statistical analysis

Graphs were generated using GraphPad Prism 8 (GraphPad Software, San Diego, CA, United States). Quantitative data are presented as the mean ± SD of three independent experiments.

## 3 Results

### 3.1 Genome mining and biosynthetic gene cluster analysis

Genome mining of *A. lappa* identified 35 putative biosynthetic gene clusters (BGCs) distributed throughout the genome, indicating substantial biosynthetic potential for the production of specialized metabolites. The predicted clusters encompassed diverse biosynthetic classes, including terpene, alkaloid-like, polyketide, phenylpropanoid/flavonoid, saccharide, cyclopeptide, fatty acid–saccharide, saccharide–alkaloid, hybrid, and unclassified pathways (Table 1). This diversity of predicted BGCs demonstrates the extensive repertoire of specialized metabolic pathways encoded within the *A. lappa* genome.

**TABLE 1:** Predicted biosynthetic gene cluster (BGC) classes identified in the *Arctium lappa* genome by plantiSMASH.

Biosynthetic class	Representative biosynthetic features identified	Putative metabolites/products
Terpene	Terpene synthases (TPS), cytochrome P450 monooxygenases, prenyltransferases, methyltransferases, dehydrogenases/reductases, acyltransferases, CoA ligases	Mono-, sesqui-, di-, and modified terpenoids
Saccharide	Glycosyltransferases, carbohydrate-modifying enzymes	Glycosylated specialized metabolites and oligosaccharides
Saccharide–Terpene (hybrid)	TPS together with glycosyltransferases and methyltransferases	Glycosylated terpene derivatives
Cyclopeptide	Peptide biosynthetic enzymes with terpene-related tailoring genes	Cyclic peptide-derived metabolites
Fatty acid–Saccharide	Fatty acid biosynthetic enzymes, desaturases, lipoxygenases, carbohydrate biosynthetic genes	Lipid-derived specialized metabolites
Saccharide–Alkaloid	Putative terpene synthase-like domains, glycosyltransferases, alkaloid-associated enzymes	Alkaloid-derived glycosides
Polyketide	Type III polyketide synthases (PKSs), oxidoreductases, methyltransferases	Polyketides and related aromatic metabolites
Phenylpropanoid/Flavonoid	Chalcone synthase-like enzymes, methyltransferases, oxidoreductases	Flavonoids, chalcones, phenolic metabolites
Alkaloid-like	Oxidoreductases, methyltransferases, amine-modifying enzymes	Putative alkaloid metabolites
Putative/Unclassified	Conserved biosynthetic enzymes lacking definitive classification	Novel or currently uncharacterized specialized metabolites

Among the identified BGCs, terpene-associated clusters were selected for further analysis because of the well-established antibacterial activities of plant terpenoids and their relevance to natural product discovery. Several terpene clusters encoded terpene synthases (TPSs) together with tailoring enzymes, including cytochrome P450 monooxygenases, methyltransferases, glycosyltransferases, prenyltransferases, dehydrogenases/reductases, acyltransferases, and CoA ligases, suggesting extensive biosynthetic diversification of terpene-derived metabolites. Representative terpene and saccharide–terpene biosynthetic gene clusters are illustrated in Figure 2.

**FIGURE 2:**
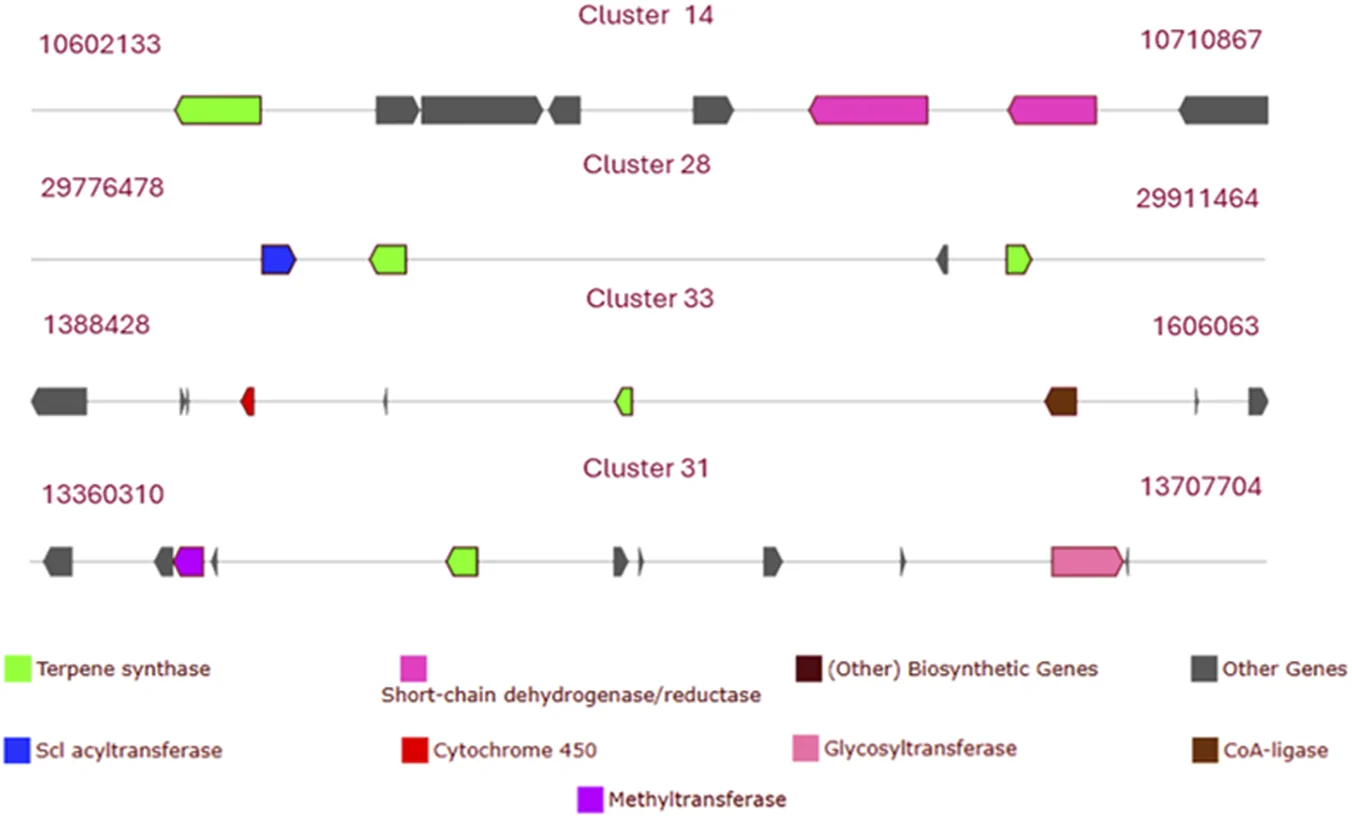
Schematic representation of terpene and saccharide–terpene biosynthetic gene clusters. Predicted biosynthetic gene clusters identified by plantiSMASH are shown as arrow diagrams, where each arrow represents an open reading frame and the orientation indicates transcriptional direction. Functional categories are distinguished by color as indicated in the figure. Clusters include: Cluster 14 (terpene, 10,602,133–10,710,867 nt): encodes a terpene synthase together with short-chain dehydrogenase/reductase genes and additional tailoring enzymes, suggesting a modified terpene product; Cluster 28 (terpene, 29,776,478–29,911,464 nt): contains a terpene synthase in association with an acyltransferase, indicating possible acylated terpene derivatives; Cluster 33 (terpene, 1,388,428–1,606,063 nt): includes a terpene synthase, cytochrome P450, and a CoA-ligase, consistent with oxidative and side-chain modifications of terpene backbones; Cluster 31 (saccharide–terpene, 13,360,310–13,707,704 nt): encodes a terpene synthase alongside glycosyltransferase and methyltransferase genes, suggesting glycosylated and methylated terpene structures. Other biosynthetic genes and non-biosynthetic neighboring genes are also displayed. Together, these clusters highlight the genomic potential for structural diversification of terpene-derived metabolites.

In addition to terpene-associated BGCs, several clusters displayed domain architectures consistent with alkaloid-, polyketide-, and phenylpropanoid/flavonoid-related biosynthesis. These clusters encoded enzymes such as type III polyketide synthases, methyltransferases, oxidoreductases, glycosyltransferases, and other biosynthetic proteins commonly associated with plant specialized metabolism. Furthermore, multiple hybrid or unclassified BGCs containing genes from different biosynthetic pathway classes were identified, suggesting the potential production of structurally diverse specialized metabolites.

Overall, the broad diversity of predicted biosynthetic gene clusters identified in the *A. lappa* genome provides a genomic resource for future functional and metabolomic investigations aimed at linking specific biosynthetic pathways with the antibacterial, biofilm inhibitory, and antibiotic-potentiating activities observed for the aqueous ethanol extract.

### 3.2 Antibacterial activity and bactericidal kinetics

The antibacterial activity of the extract was evaluated against six ESKAPE-like bacterial strains by determining the minimum inhibitory concentration (MIC) and minimum bactericidal concentration (MBC) (Table 2). The extract exhibited broad-spectrum antibacterial activity, with MIC values ranging from 64 to 512 μg/mL and MBC values ranging from 128 to 512 μg/mL. Using the above crude-extract cutoffs, the MIC of 64 μg/mL against *P. aeruginosa* falls within the significant-activity range, whereas MICs of 128–512 μg/mL against the remaining strains fall within the moderate-activity range.

**TABLE 2:** Minimum inhibitory concentration (MIC), minimum bactericidal concentration (MBC) and minimum biofilm inhibitory concentration (MBIC_50_) of *Arctium lappa* extract against ESKAPE-like bacterial strains.

Bacteria	MIC (µg/mL)	MBC (µg/mL)	MBIC_50_ (µg/mL)
*Staphylococcus aureus* ATCC 25923	128	256	256
*Pseudomonas aeruginosa* ATCC 27853	64	256	128
*Enterobacter* sp. RIT 637	256	512	128
*Enterococcus faecium* RIT 857	512	512	128
*Klebsiella aerogenes* RIT 3131	256	256	128
*Acinetobacter* sp. RIT 587	128	128	64

Among the tested strains, *P. aeruginosa* ATCC 27853 exhibited the greatest susceptibility, with the lowest MIC value (64 μg/mL), although a four-fold higher concentration (256 μg/mL) was required to achieve bactericidal activity. *Acinetobacter* sp. RIT587 and *S. aureus* ATCC 25923 displayed MIC values of 128 μg/mL; however, only *Acinetobacter* sp. exhibited identical MIC and MBC values (128 μg/mL). Intermediate susceptibility was observed for *Enterobacter* sp. RIT637 and *K. aerogenes* RIT3131, both of which exhibited MIC values of 256 μg/mL. Notably, *K. aerogenes* demonstrated complete killing at the same concentration, whereas *Enterobacter* sp. required a two-fold higher concentration (512 μg/mL) for bactericidal activity. *Enterococcus faecium* RIT857 was the least susceptible strain, exhibiting MIC and MBC values of 512 μg/mL.

To further characterize the bactericidal activity of the extract, time-kill assays were performed using the previously determined MBC values for each strain. The extract exhibited rapid and sustained antibacterial activity against all tested organisms. Gram-negative bacteria, including *P. aeruginosa*, *Acinetobacter* sp., *Enterobacter* sp., and *K. aerogenes*, exhibited a ≥3-log_10_ reduction in viable cell counts within 6 h of treatment. In contrast, the Gram-positive organisms *E. faecium* and *S. aureus* displayed slower killing kinetics, reaching bactericidal thresholds after 12 h and 12–24 h, respectively (Figure 3).

**FIGURE 3:**
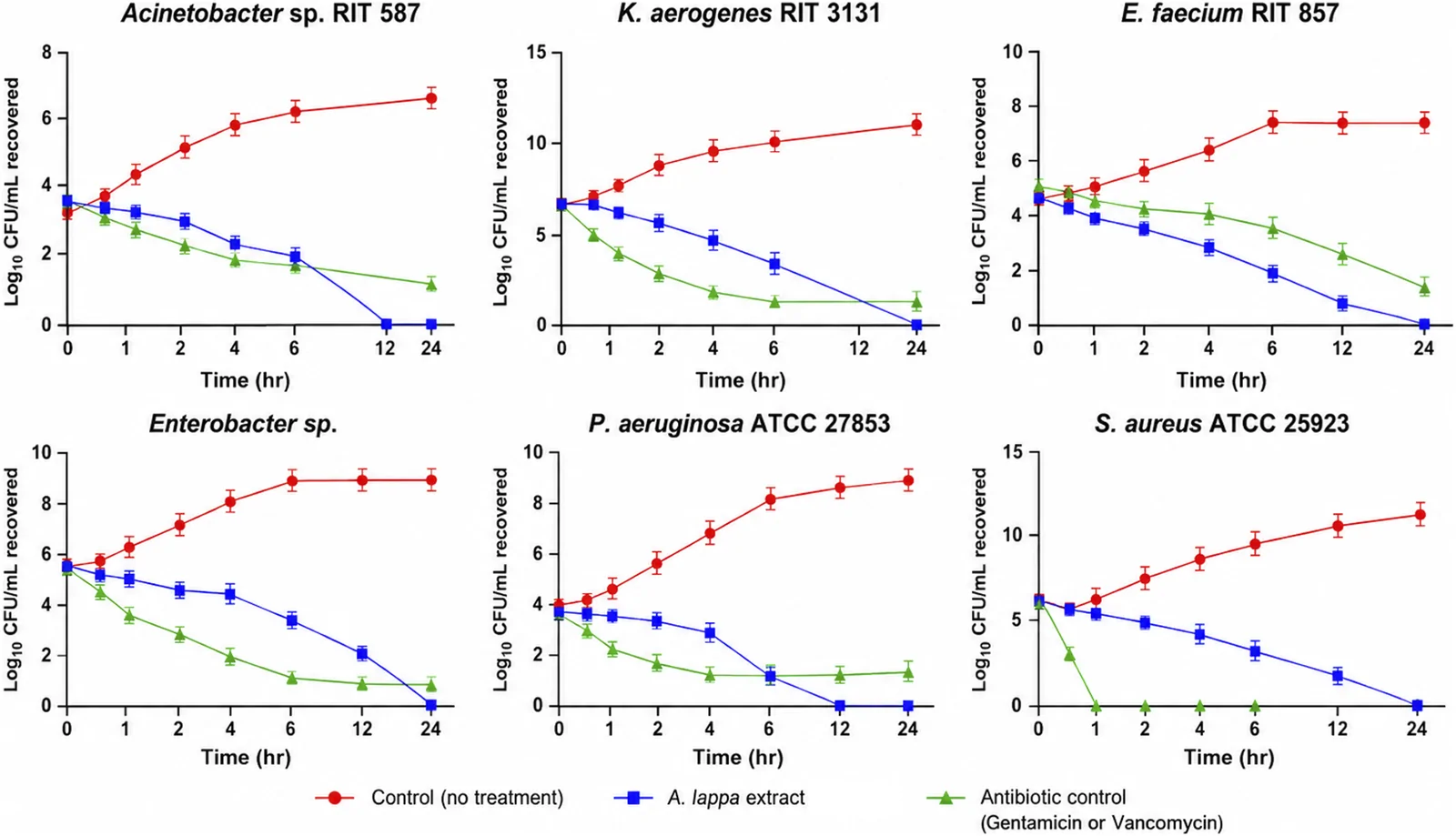
Time-kill kinetics of the 80% aqueous ethanol extract of *Arctium lappa* against representative members of the ESKAPE group. *Enterococcus faecium* RIT857, *Staphylococcus aureus* ATCC 25923, *Klebsiella aerogenes* RIT3131, *Acinetobacter* sp. RIT587, *Pseudomonas aeruginosa* ATCC 27853, and *Enterobacter* sp. RIT637 were exposed to the 80% aqueous ethanol extract of *A. lappa* at the previously determined MBCs for 24 h. Viable bacterial counts were determined at the indicated time points by plating serial dilutions on Mueller–Hinton agar and expressed as log_10_ CFU/mL recovered. Untreated cultures served as growth controls, while gentamicin (Gram-negative bacteria) or vancomycin (Gram + bacteria) each tested at their respective MBCs served as antibiotic controls. Data are presented as mean ± SD from three independent experiments.

No viable colonies were detected after 24 h of treatment for most strains, whereas untreated controls exhibited continued growth throughout the experiment.

### 3.3 Biofilm inhibition and morphological alterations induced by *Arctium lappa* extract

The ability of the 80% aqueous ethanol extract of *A*. *lappa* to inhibit biofilm formation was evaluated against six ESKAPE-like bacterial strains using a crystal violet biofilm assay. Biofilm biomass decreased in a concentration-dependent manner for all tested strains (Figure 4). The concentration required to inhibit 50% of biofilm formation (MBIC_50_) varied among the bacterial species (Table 2). *Acinetobacter* sp. RIT587 was the most susceptible strain, exhibiting a MBIC_50_ of 64 μg/mL, whereas *S. aureus* ATCC 25923 exhibited the lowest susceptibility, requiring 256 μg/mL to achieve equivalent inhibition. Intermediate susceptibility was observed for *P. aeruginosa* ATCC 27853, *Enterobacter* sp. RIT637, *E. faecium* RIT857, and *K. aerogenes* RIT 3131, all of which exhibited MBIC_50_ values of 128 μg/mL. A substantial reduction in biofilm biomass was observed at concentrations ≥128 μg/mL for most strains, with the greatest inhibition occurring at the highest concentrations tested.

**FIGURE 4:**
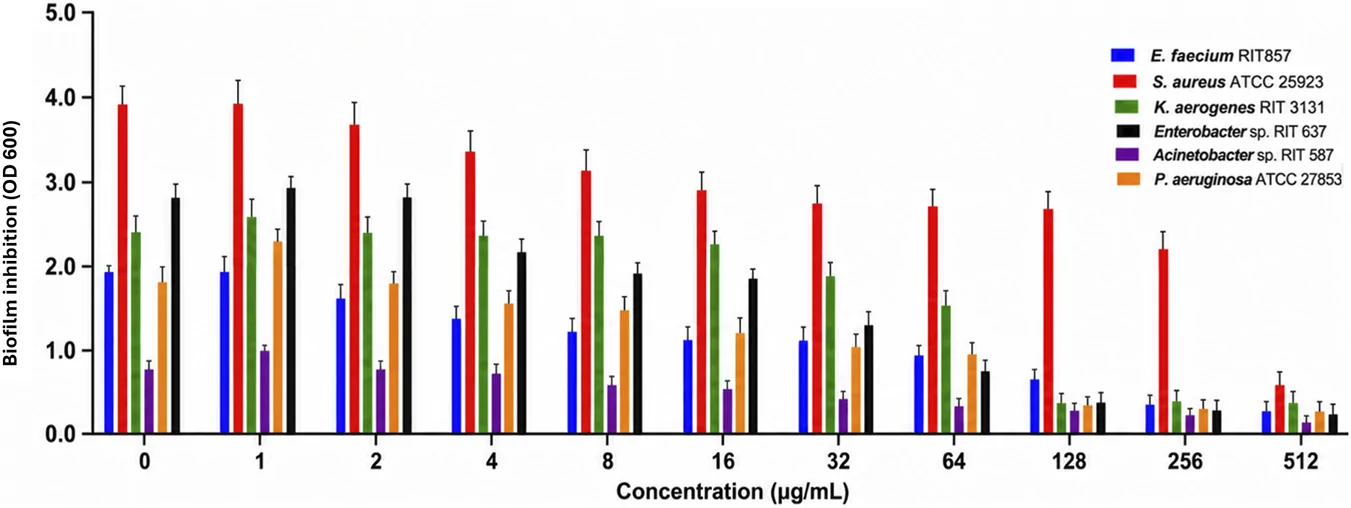
Concentration-dependent inhibition of biofilm formation by the 80% aqueous ethanol extract of *Arctium lappa*. Biofilm formation by *Enterococcus faecium* RIT 857, *Staphylococcus aureus* ATCC 25923, *Klebsiella aerogenes* RIT3131, *Acinetobacter* sp. RIT587, *Pseudomonas aeruginosa* ATCC 27853, and *Enterobacter* sp. RIT 637 was quantified using the crystal violet assay following 24 h of incubation with increasing concentrations of the extract (0–512 μg/mL). Biofilm biomass was determined by measuring absorbance at OD_600_ after crystal violet staining and ethanol solubilization. Data are presented as mean ± SD from three independent experiments.

To further investigate the effects of the extract on bacterial structure, scanning electron microscopy (SEM) was performed on treated and untreated cells. Untreated bacteria exhibited normal cellular morphology, characterized by smooth surfaces, intact cell envelopes, and well-defined cellular architecture. In contrast, extract-treated cells displayed marked morphological alterations, including surface irregularities, membrane deformation, cellular collapse, and disruption of biofilm architecture (Figure 5). These structural changes were observed in both *P. aeruginosa* and *S. aureus*, supporting the biofilm inhibitory activity observed in the crystal violet assays.

**FIGURE 5:**
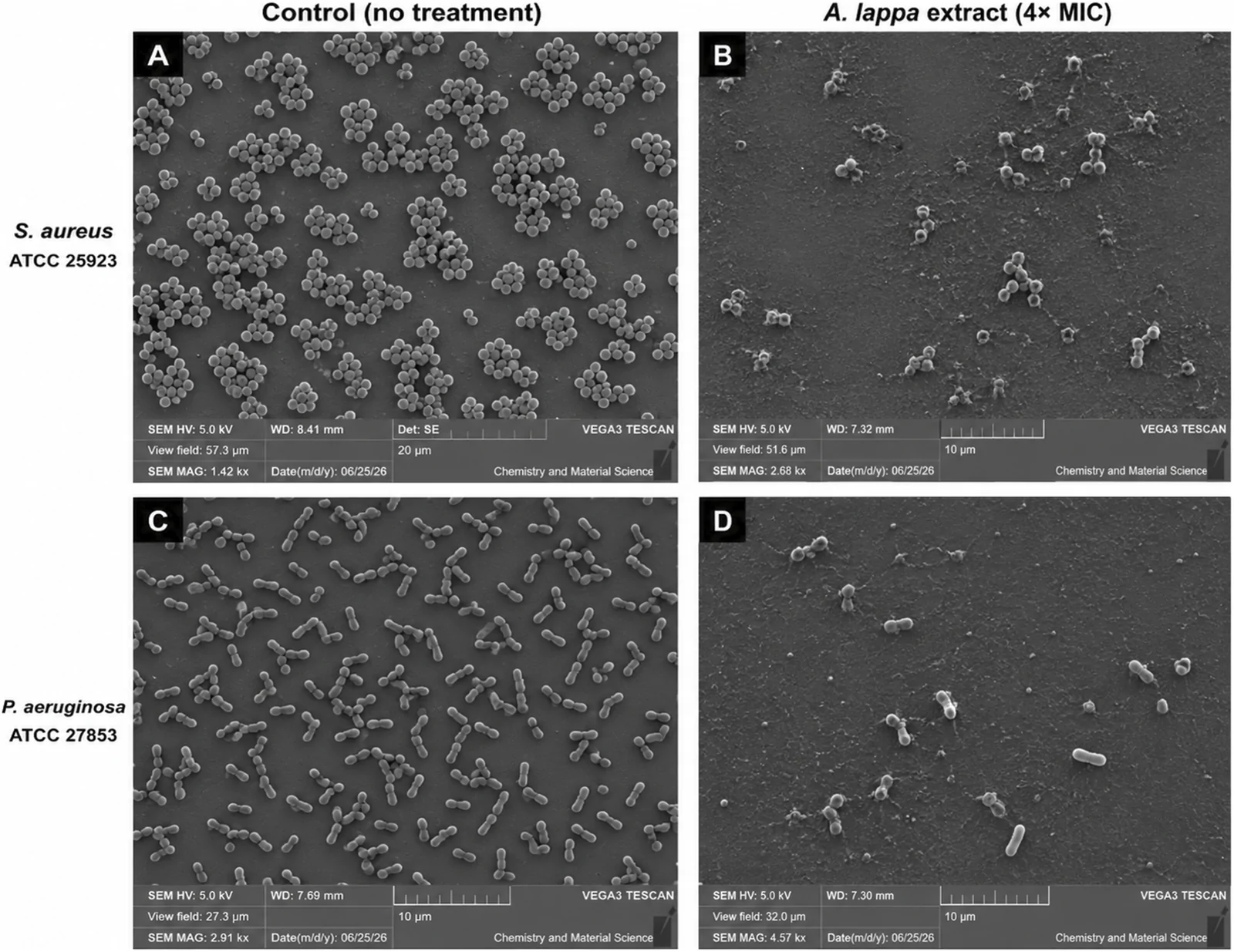
Scanning electron microscopy (SEM) analysis of bacterial morphological changes following treatment with the 80% aqueous ethanol extract of *Arctium lappa*. Representative SEM micrographs of *Staphylococcus aureus* ATCC 25923 **(A,B)** and *Pseudomonas aeruginosa* ATCC 27853 **(C,D)** are shown. Untreated cells **(A,C)** exhibited intact morphology with smooth, well-defined cell surfaces and typical cellular organization. In contrast, cells exposed to the *A. lappa* extract at 4× MIC for 2 h **(B,D)** displayed marked morphological alterations, including reduced cell density, surface deformation, membrane disruption, cell shrinkage, and structural collapse, consistent with extract-induced cellular damage. Images are representative of three independent experiments.

Atomic force microscopy (AFM) analysis further characterized the surface morphological changes induced by the *A. lappa* extract (Figure 6). Untreated *P. aeruginosa* ATCC 27853 and *S. aureus* ATCC 25923 cells exhibited relatively smooth surface topography and well-defined cellular morphology. Following treatment with the *A. lappa* extract, both species displayed increased surface roughness, irregular surface contours, and pronounced topographical alterations. In *P. aeruginosa*, treated cells exhibited prominent grooves, depressions, and disruption of the normally uniform surface architecture compared with untreated controls. Similarly, treated *S. aureus* cells displayed irregular surface morphology and deformation of the cell envelope, resulting in a less organized surface topography. These alterations were consistently observed in treated cells and were absent in untreated controls.

**FIGURE 6:**
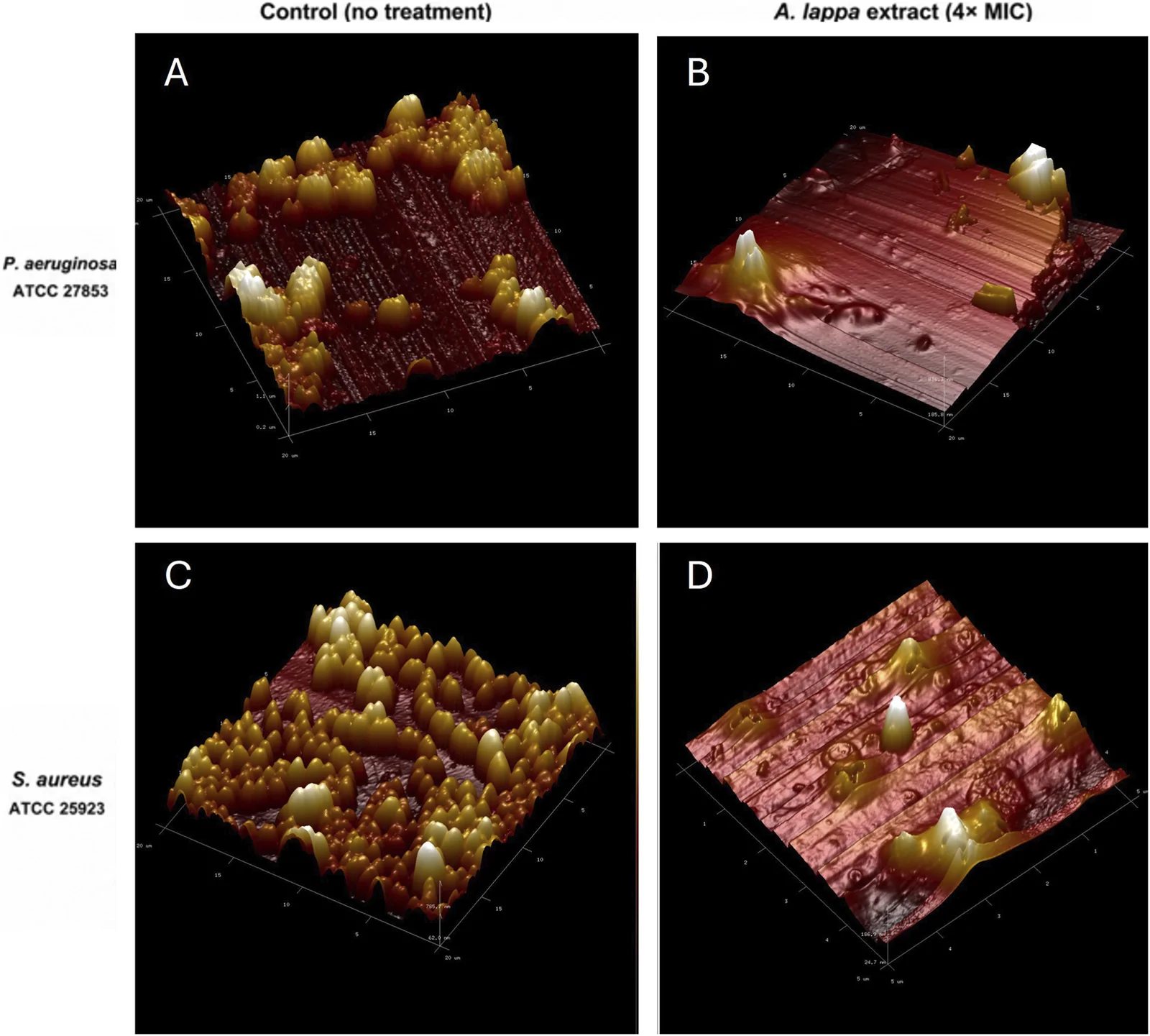
Atomic force microscopy (AFM) analysis of bacterial morphology before and after treatment with *Arctium lappa* extract. **(A)** Untreated *Pseudomonas aeruginosa* ATCC 27853; **(B)** *P. aeruginosa* treated with *A. lappa* extract; **(C)** untreated *Staphylococcus aureus* ATCC 25923; and **(D)** *S. aureus* treated with *A. lappa* extract. Treated cells exhibited marked surface irregularities, increased roughness, and membrane damage compared with untreated controls, consistent with membrane disruption as a potential antibacterial mechanism of the extract.

Together, the crystal violet biofilm assay, SEM, and AFM analyses consistently showed reduced biofilm formation and pronounced alterations in bacterial surface morphology following treatment with the *A. lappa* extract.

### 3.4 Membrane damage and oxidative stress induced by *Arctium lappa* extract

To investigate the effects of the extract on bacterial membrane integrity and intracellular reactive oxygen species (ROS) production, LIVE/DEAD fluorescence staining and H_2_DCFDA-based ROS assays were performed using *P. aeruginosa* ATCC 27853 and *S. aureus* ATCC 25923.

Membrane integrity was assessed using LIVE/DEAD fluorescence staining with SYTO 9 and propidium iodide (PI) (Figure 7). Untreated control cells exhibited predominantly green fluorescence, indicating intact cell membranes and high cell viability. In contrast, cells treated with the *A. lappa* extract at 4× MIC for 2 h exhibited a marked increase in red fluorescence, indicating increased PI uptake and reduced membrane integrity. This pattern was observed in both bacterial species, with a greater proportion of red-fluorescent cells observed following treatment compared with untreated controls.

**FIGURE 7:**
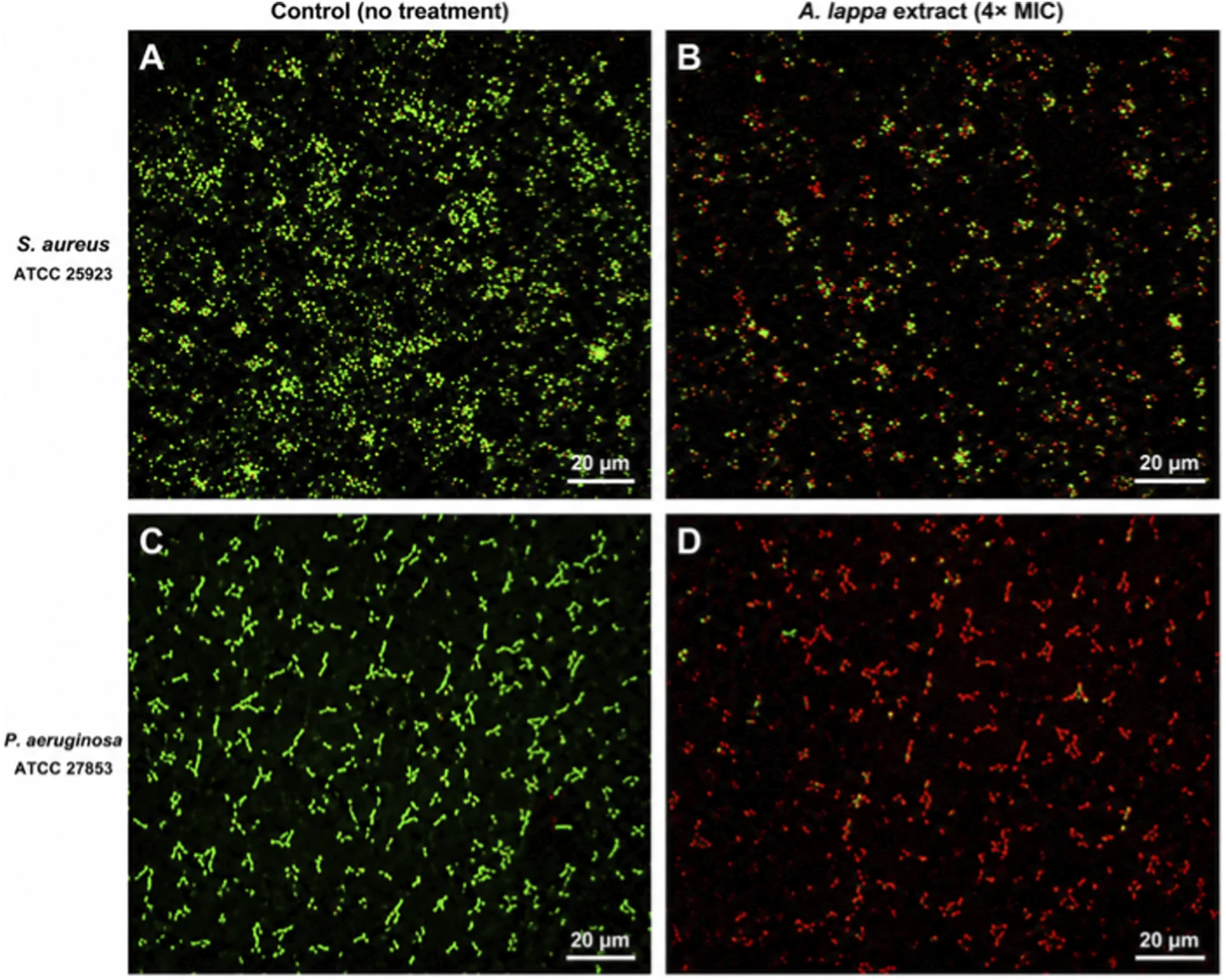
Live/dead fluorescence microscopy of *Staphylococcus aureus* ATCC 25923 and *Pseudomonas aeruginosa* ATCC 27853 following treatment with *Arctium lappa* extract. Representative merged fluorescence images obtained using the LIVE/DEAD BacLight bacterial viability assay. Live bacteria stained with SYTO 9 are shown in green, whereas membrane-compromised (dead) bacteria stained with propidium iodide (PI) are shown in red. **(A)** *S. aureus* untreated control, **(B)** *S. aureus* treated with *A. lappa* extract (4 × MIC), **(C)** *P. aeruginosa* untreated control, and **(D)** *P. aeruginosa* treated with *A. lappa* extract (4 × MIC). Images were acquired from the same microscopic field using the SYTO 9 and PI filter sets and digitally merged. Treatment with the extract resulted in a marked increase in PI-positive cells and a concomitant reduction in SYTO 9-positive cells compared with untreated controls, indicating disruption of bacterial membrane integrity and increased cell death. Scale bars = 20 μm.

Intracellular ROS production was quantified using the fluorescent probe H_2_DCFDA (Figure 8). Treatment with the *A. lappa* extract at 4× MIC increased normalized intracellular ROS levels in both bacterial species compared with untreated and vehicle controls. In *S. aureus* ATCC 25923, normalized ROS levels increased from 100% ± 6% in untreated cells to 181% ± 11% following extract treatment, whereas the vehicle control remained comparable to untreated cells (103% ± 5%). Similarly, normalized ROS levels in *P. aeruginosa* ATCC 27853 increased from 100% ± 6% in untreated cells to 214% ± 13% following treatment with the extract, while the vehicle control showed no appreciable change (97% ± 5%).

**FIGURE 8:**
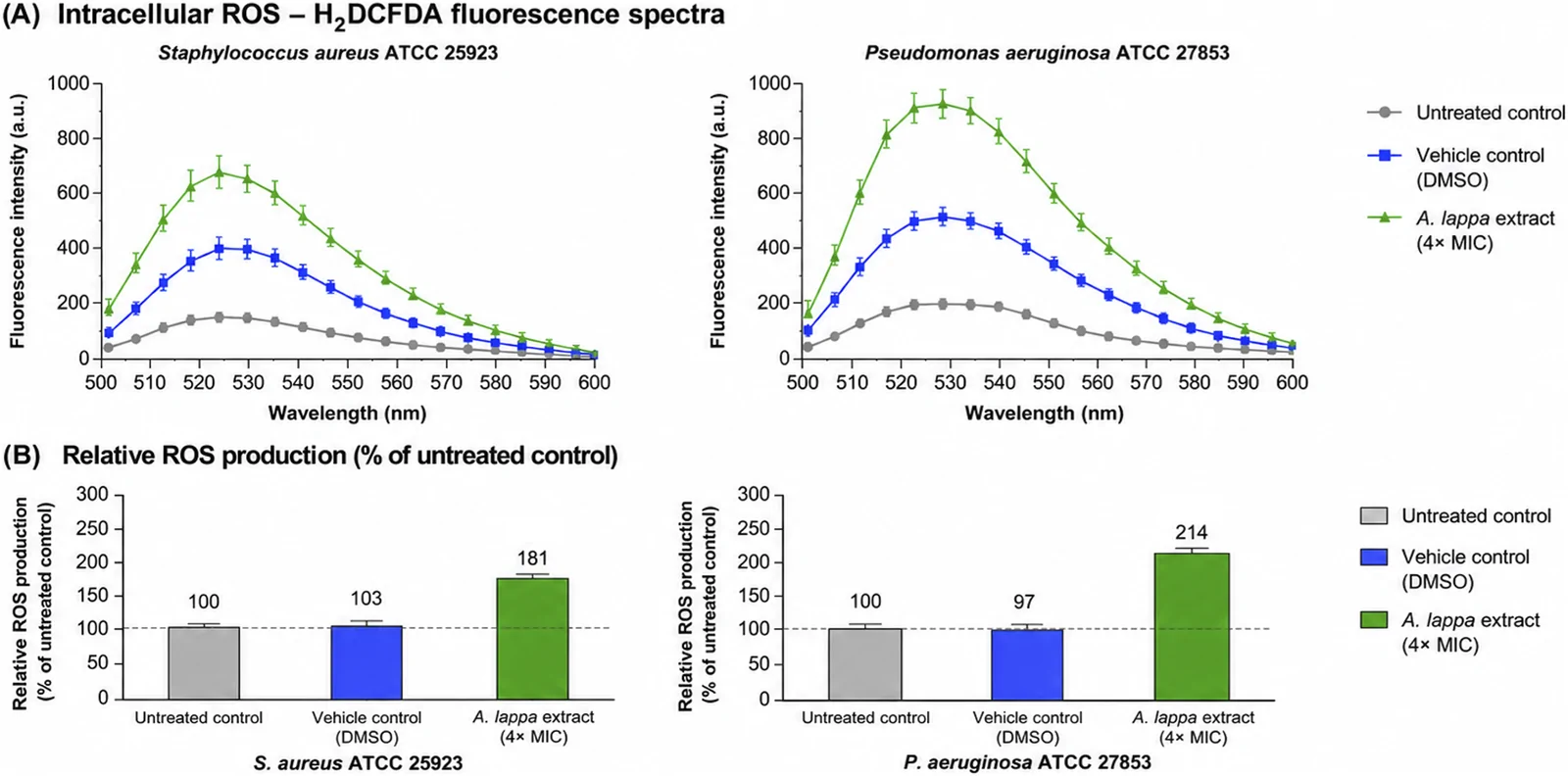
Intracellular reactive oxygen species (ROS) production in *Staphylococcus aureus* ATCC 25923 and *Pseudomonas aeruginosa* ATCC 27853 following treatment with the 80% aqueous ethanol extract of *Arctium lappa*. **(A)** H_2_DCFDA fluorescence emission spectra of untreated cells, vehicle control (DMSO)-treated cells, and cells treated with the *A. lappa* extract at 4× MIC for 2 h. Fluorescence emission spectra were recorded using an excitation wavelength of 485 nm and an emission scan from 500 to 600 nm. **(B)** Relative intracellular ROS production following treatment with the *A. lappa* extract. Fluorescence intensity was normalized to the corresponding OD_600_ values and expressed as a percentage of the untreated control. Bars represent the mean ± SD of three independent experiments.

Together, the LIVE/DEAD staining and H_2_DCFDA assays showed increased membrane permeability and intracellular ROS accumulation in both bacterial species following treatment with the aqueous ethanol extract of *A. lappa* (Figures 7, 8).

### 3.5 Effects of *Arctium lappa* extract on the activity of conventional antibiotics

The effect of the aqueous ethanol extract of *A. lappa* on antibiotic activity was evaluated using a modified disk diffusion assay against six ESKAPE-like bacterial strains. Inhibition zone diameters were compared between antibiotic disks alone and disks supplemented with the extract (Table 3).

**TABLE 3:** Effect of *Arctium lappa* extract on antibiotic susceptibility: zone shifts and interaction classification.

Pathogen	Antibiotic (µg)	Control (mm)	Extract (mm)	Observed effect (mm)	Interaction
*S. Aureus*	Tetracycline (TE 30)	22.6	22.6	0	Indifferent
*S. Aureus*	Trimethoprim/Sulfamethoxazole (SXT 25)	21.3	21.51	0.21	Indifferent
*S. Aureus*	Vancomycin (VA 30)	15.58	16.37	0.79	Synergistic
*S. Aureus*	Chloramphenicol (C 30)	19.6	20.73	1.13	Synergistic
*S. Aureus*	Rifampin (RD 5)	24.67	25.71	1.04	Synergistic
*P. Aeruginosa*	Rifampin (RD 5)	35.99	35.8	−0.19	Indifferent
*P. Aeruginosa*	Clindamycin (DA 2)	18.26	19.8	1.54	Synergistic
*P. Aeruginosa*	Kanamycin (K 30)	16.2	16.21	0.01	Indifferent
*P. Aeruginosa*	Polymyxin B (PB 300)	7.57	7.37	−0.2	Indifferent
*P. Aeruginosa*	Colistin (CT 10)	6.31	6.51	0.2	Indifferent
*K. Aerogenes*	Rifampin (RD 5)	7.45	7.5	0.05	Indifferent
*K. Aerogenes*	Tetracycline (TE 30)	21.55	22.8	1.25	Synergistic
*K. Aerogenes*	Nitrofurantoin (N 30)	22.15	23.01	0.86	Synergistic
*K. Aerogenes*	Polymyxin B (PB 300)	12.73	11.63	−1.1	Antagonistic
*K. Aerogenes*	Colistin (CT 10)	11.23	11.18	−0.05	Indifferent
*Enterobacter sp·*	Trimethoprim/Sulfamethoxazole (SXT 25)	27.41	28.1	0.69	Synergistic
*Enterobacter sp·*	Kanamycin (K 30)	22.93	19.36	−3.57	Antagonistic
*Enterobacter sp·*	Tetracycline (TE 30)	24.4	21.52	−2.88	Antagonistic
*Enterobacter sp*	Polymyxin B (PB 300)	13.65	13.84	0.19	Indifferent
*Enterobacter sp·*	Colistin (CT 10)	11.97	12.21	0.24	Indifferent
*E. Faecium*	Vancomycin (VA 30)	17.71	19.1	1.39	Synergistic
*E. Faecium*	Chloramphenicol (C 30)	27.27	30.24	2.97	Synergistic
*E. Faecium*	Rifampin (RD 5)	34.4	35.15	0.75	Synergistic
*E. Faecium*	Tetracycline (TE 30)	30.94	32.68	1.74	Synergistic
*E. Faecium*	Streptomycin (S 10)	14.99	16.52	1.53	Synergistic
*Acinetobacter sp·*	Rifampin (RD 5)	30.98	35.97	4.99	Synergistic
*Acinetobacter sp·*	Colistin (CT 10)	6.3	6.4	0.1	Indifferent
*Acinetobacter sp·*	Tetracycline (TE 30)	35.26	33.36	−1.89	Antagonistic
*Acinetobacter sp·*	Nitrofurantoin (N 30)	19.11	19.69	0.58	Synergistic
*Acinetobacter sp·*	Polymyxin B (PB 300)	6.78	6.69	−0.09	Indifferent

The magnitude of the interaction varied among the bacterial species and antibiotics tested. The largest increase in inhibition zone diameter was observed for *Acinetobacter* sp. RIT587 in combination with rifampin (+4.99 mm). Additional increases were observed for *E. faecium* RIT857 in combination with chloramphenicol (+2.97 mm), tetracycline (+1.74 mm), streptomycin (+1.53 mm), and vancomycin (+1.39 mm).

In *P. aeruginosa* ATCC 27853, the greatest increase in inhibition zone diameter was observed with clindamycin (+1.54 mm), whereas only minor changes were detected for kanamycin, colistin, polymyxin B, and rifampin. Similarly, *K. aerogenes* RIT3131 exhibited increased inhibition zone diameters in combination with tetracycline (+1.25 mm) and nitrofurantoin (+0.86 mm), while only small changes were observed for rifampin and colistin.

For *S. aureus* ATCC 25923, increased inhibition zone diameters were observed in combination with chloramphenicol (+1.13 mm), rifampin (+1.04 mm), and vancomycin (+0.79 mm), whereas no measurable change was detected with tetracycline. In contrast, *Enterobacter* sp. RIT637 exhibited both increases and decreases in inhibition zone diameter depending on the antibiotic tested. Increases were observed for trimethoprim-sulfamethoxazole (+0.69 mm), polymyxin B (+0.19 mm), and colistin (+0.24 mm), whereas decreases were observed for kanamycin (−3.57 mm) and tetracycline (−2.88 mm). Negative interactions were also observed for *Acinetobacter* sp. in combination with tetracycline (−1.89 mm) and for *K. aerogenes* in combination with polymyxin B (−1.10 mm).

Overall, increases and decreases in inhibition zone diameters were observed depending on the antibiotic–bacterium combination evaluated (Table 3).

## 4 Discussion

Genome-guided natural product discovery has emerged as a powerful strategy for identifying novel antimicrobial agents in response to the increasing global burden of antimicrobial resistance (Newman and Cragg, 2020; Navarro-Muñoz et al., 2020). Medicinal plants have emerged as promising sources of antibacterial metabolites against clinically important and multidrug-resistant pathogens (Majeed et al., 2022; Hasan, 2025). Although medicinal plants have long been investigated as sources of bioactive metabolites, most studies have relied primarily on phenotypic screening without considering the genomic basis underlying specialized metabolite biosynthesis (Atanasov et al., 2021; Dias et al., 2012; Newman and Cragg, 2020). Recent advances in whole-genome sequencing and biosynthetic gene cluster prediction have enabled the integration of genomic information with functional analyses, providing a powerful framework for prioritizing candidate biosynthetic pathways and accelerating natural product discovery (Navarro-Muñoz et al., 2020; Atanasov et al., 2021; Newman and Cragg, 2020).

In the present study, genome mining of *A. lappa* identified 35 putative biosynthetic gene clusters (BGCs), including terpene-, polyketide-, phenylpropanoid/flavonoid-, alkaloid-like-, saccharide-, and hybrid-associated pathways. The diversity of these predicted clusters indicates substantial biosynthetic potential and provides a genomic framework supporting the production of structurally diverse specialized metabolites with antimicrobial activity. In particular, the identification of multiple terpene-associated BGCs encoding terpene synthases together with cytochrome P450 monooxygenases, glycosyltransferases, methyltransferases, and other tailoring enzymes suggests an extensive capacity for terpenoid diversification, a feature commonly associated with bioactive plant secondary metabolites (Huang et al., 2022).

The first chromosome-level genome assembly of *A. lappa* was reported by Yang et al. (2022), who generated a high-quality reference genome for a cultivated Chinese accession and primarily focused on genome assembly, comparative genomics, and genes involved in lignan biosynthesis and plant development. Their work identified more than 32,000 protein-coding genes and established an important genomic resource for future studies of *A. lappa* (Yang et al., 2022). More recently, Feng et al. (2025) generated a second chromosome-level genome assembly from the Chinese cultivar ‘Peixian yellow’, improving genomic resources for the species and investigating carotenoid biosynthesis. Comparative analyses between Chinese accessions revealed extensive genomic variation, including millions of single nucleotide polymorphisms (SNPs), hundreds of thousands of insertion/deletion polymorphisms (INDELs), and thousands of structural variants, demonstrating considerable genetic diversity within *A. lappa* (Feng et al., 2025).

Unlike these previous studies, which primarily emphasized genome assembly quality, comparative genomics, and metabolic pathways related to plant development or phytochemical accumulation, the present study focused on the biosynthetic potential associated with antimicrobial metabolites. Furthermore, the *A. lappa* investigated here represents a population naturalized in the United States rather than a cultivated Chinese accession. Geographic separation and adaptation to distinct environmental conditions may contribute to genomic and metabolic diversification, potentially influencing the composition and regulation of specialized metabolite biosynthetic pathways (Feng et al., 2025). Although comparative genomic analyses between geographically distinct populations were beyond the scope of the present study, the genome reported here expands the available genomic resources for *A. lappa* and provides a valuable foundation for future population genomic, metabolomic, and evolutionary investigations.

An important strength of the present study is the integration of genome mining with comprehensive biological validation. Rather than relying solely on genomic predictions, the predicted biosynthetic potential was evaluated alongside antibacterial susceptibility testing, bactericidal kinetics, biofilm inhibition, membrane integrity assays, intracellular ROS production, scanning electron microscopy, atomic force microscopy, and antibiotic interaction analyses. This integrated strategy enables comparison between predicted biosynthetic capacity and experimentally observed antibacterial phenotypes, facilitating the prioritization of candidate metabolite classes for future isolation and characterization. Among the predicted biosynthetic pathways, terpene-associated clusters are of particular interest because plant terpenoids have been widely reported to possess antibacterial, biofilm inhibitory, membrane-disruptive, and antibiotic-potentiating activities (Huang et al., 2022). Similarly, polyketides, phenylpropanoids, flavonoids, and alkaloids constitute important classes of antimicrobial natural products capable of affecting bacterial membranes, quorum sensing, oxidative stress, and multiple intracellular targets (Atanasov et al., 2021; Newman and Cragg, 2020). Therefore, the broad diversity of biosynthetic pathways identified in the *A. lappa* genome is consistent with the broad-spectrum antibacterial and mechanistic activities observed for the aqueous ethanol extract in the present study. Collectively, these findings demonstrate the value of integrating genome mining with functional microbiological analyses to facilitate the discovery of plant-derived antimicrobial metabolites and provide a foundation for future isolation and characterization of the bioactive metabolites responsible for the observed antibacterial activity.

The aqueous ethanol extract of *A. lappa* exhibited broad-spectrum antibacterial activity against all six ESKAPE-like bacterial strains tested, with MIC values ranging from 64 to 512 μg/mL. Similar antibacterial activity has previously been reported for *A. lappa* extracts against clinically relevant bacterial pathogens, supporting the antimicrobial potential of this species (Bahramian et al., 2023; Wang et al., 2019). Among the organisms evaluated, *P. aeruginosa* exhibited the greatest susceptibility, whereas *E*. *faecium* was the least susceptible. Time-kill analyses further demonstrated rapid bactericidal activity, particularly against the Gram-negative pathogens, which exhibited a ≥3-log_10_ reduction in viable cell counts within 6 h of treatment. This susceptibility pattern differs from that commonly reported for many plant-derived extracts, in which Gram-positive bacteria are often more susceptible because the outer membrane of Gram-negative bacteria restricts the diffusion of hydrophobic phytochemicals (Guimarães et al., 2019).

Biofilm formation is a major contributor to antimicrobial tolerance and persistence among ESKAPE pathogens. The aqueous ethanol extract markedlyinhibited biofilm formation in all strains tested, with *Acinetobacter* sp. exhibiting the greatest susceptibility. Similar biofilm inhibitory activity has previously been reported for *A. lappa* leaf fractions against *S*. *aureus* (Tang et al., 2014). The broad-spectrum biofilm inhibitory activity observed in this study may be associated with metabolites predicted by genome mining, including terpenoids, flavonoids, and polyketide-derived metabolites. However, these metabolites were not experimentally identified in the extract. These metabolite classes have been widely reported to interfere with quorum sensing, bacterial adhesion, extracellular polymeric substance production, and biofilm maturation (El et al., 2023). The agreement between genomic predictions and the observed phenotypic activity further supports the value of genome-guided approaches for prioritizing medicinal plants with antibacterial and biofilm inhibitory potential.

Mechanistic analyses provided additional insight into the antibacterial mode of action of the extract. SEM and AFM consistently demonstrated increased surface roughness, irregular surface topology, membrane deformation, and partial collapse of bacterial cells following treatment, suggesting that membrane disruption may contribute to the antibacterial activity of the extract. These observations were further supported by LIVE/DEAD fluorescence staining, which demonstrated a marked increase in propidium iodide-positive cells following treatment, indicating compromised membrane integrity. Similar membrane-associated damage has been reported for terpene-rich plant extracts and essential oil constituents, which interact directly with bacterial envelopes and increase membrane permeability (Guimarães et al., 2019; Yap et al., 2014). These effects were particularly evident in *P. aeruginosa*, suggesting that components of the extract may partially overcome the permeability barrier characteristic of Gram-negative bacteria.

In addition to membrane disruption, treatment with the extract induced a pronounced increase in intracellular reactive oxygen species (ROS) production in both *P. aeruginosa* and *S. aureus*. Oxidative stress has increasingly been recognized as an important contributor to bacterial killing by natural antimicrobial metabolites and may act synergistically with membrane damage to accelerate cell death (Gupta et al., 2021). The simultaneous observation of increased ROS production, membrane permeabilization, and extensive structural damage suggests that multiple cellular processes may contribute to the antibacterial activity of the extract rather than indicating a single mechanism of action. Although the SEM, AFM, LIVE/DEAD fluorescence staining, and ROS assays consistently support membrane damage and oxidative stress following treatment, these observations do not establish definitive molecular mechanisms of action. Additional molecular and biochemical studies will be required to confirm the pathways involved. Such multimodal mechanisms are particularly attractive because they may reduce the likelihood of resistance development while enhancing antibacterial efficacy.

The enhanced activity observed against Gram-negative bacteria in the present study may reflect the combined action of multiple classes of specialized metabolites extracted with 80% aqueous ethanol, including terpenoids, phenylpropanoids, flavonoids, and other polar and moderately lipophilic classes of specialized metabolites predicted through genome mining. Such combinations may facilitate membrane permeabilization, oxidative stress induction, and disruption of bacterial physiology, consistent with the membrane damage, increased ROS production, and rapid bactericidal activity observed in this study. Similar membrane-disruptive and anti-virulence effects have recently been reported for arctigenin, a lignan isolated from *A. lappa*, against *P. aeruginosa*, further supporting the ability of *Arctium*-derived metabolites to target Gram-negative pathogens (Koshak et al., 2024). However, additional phytochemical characterization will be required to identify the specific metabolites responsible for the antibacterial activity of the crude extract.

An additional finding of this study was the ability of the extract to potentiate the activity of selected conventional antibiotics. The largest increase in inhibition zone diameter was observed for rifampin against *Acinetobacter* sp., while chloramphenicol, tetracycline, and streptomycin also exhibited enhanced activity against *E. faecium*. Similar antibiotic-potentiating effects have previously been reported for *A. lappa* extracts and isolated burdock-derived metabolites (Guedes et al., 2024; Lewis et al., 2024). Although checkerboard assays and fractional inhibitory concentration index (FICI) analyses were not performed, the observed increases in inhibition zone diameter suggest that components of the extract may facilitate antibiotic activity. Increased membrane permeability, disruption of bacterial defense mechanisms, and enhanced intracellular antibiotic accumulation have all been proposed as mechanisms through which plant-derived terpenes potentiate antibiotic efficacy (Bardaji et al., 2025b; Guimarães et al., 2019).

Several limitations of this study should be acknowledged. First, the antibacterial activity was evaluated using a crude aqueous ethanol extract; therefore, the individual metabolites responsible for the observed biological effects remain unidentified. Secondly, although genome mining identified 35 putative biosynthetic gene clusters, neither their expression nor the production of their corresponding metabolites was experimentally validated. Consequently, direct associations between specific biosynthetic pathways and the observed antibacterial phenotypes cannot yet be established. Finally, all experiments were conducted *in vitro*, and additional studies are required to evaluate efficacy, toxicity, pharmacological properties, and therapeutic potential in biologically relevant *in vivo* models. No GC-MS, LC-MS, HPLC, NMR, or other metabolite-fingerprinting analysis was performed on the tested extract; accordingly, the study does not assign the observed antibacterial or biofilm-inhibitory effects to specific chemical constituents.

Future studies should therefore focus on bioassay-guided fractionation, metabolomic characterization, and molecular networking approaches to identify the metabolites responsible for the antibacterial activity of *A. lappa*. Integrating metabolomic analyses with genome mining will facilitate direct associations between biosynthetic gene clusters and their corresponding bioactive metabolites, providing a powerful platform for natural product discovery. In addition, transcriptomic analyses, checkerboard assays, resistance-development studies, and *in vivo* infection models will further clarify the mechanisms of action, therapeutic potential, and clinical applicability of *A. lappa*-derived metabolites as novel antimicrobial agents or antibiotic adjuvants.

Overall, the convergence of genome mining, antibacterial susceptibility testing, time-kill kinetics, biofilm inhibition assays, microscopy, ROS detection, and antibiotic interaction testing demonstrates that the aqueous ethanol extract of *A. lappa* exerts antibacterial and biofilm-inhibitory effects associated with membrane damage and increased oxidative stress. Because the extract was not chemically profiled, these findings cannot be attributed to specific metabolites or directly linked to individual predicted biosynthetic gene clusters. The results provide a basis for future bioassay-guided fractionation and metabolomic characterization.

## 5 Conclusion

The present study demonstrates antibacterial and biofilm-inhibitory activity of an aqueous ethanol extract of *A. lappa* against a panel of ESKAPE-like bacteria. Genome mining identified 35 putative biosynthetic gene clusters, while microbiological and cell-based assays showed bactericidal activity, altered membrane integrity, increased intracellular ROS, and changes in the activity of selected antibiotics. These observations support further investigation of *A. lappa* as a source of antibacterial natural products; however, chemical profiling and bioassay-guided isolation are required before the active metabolites, and their molecular mechanisms can be established.

## Data Availability

The datasets presented in this study can be found in online repositories. The names of the repository/repositories and accession number(s) can be found below: https://www.ncbi.nlm.nih.gov/genbank/, Bioproject Number PRJNA1167729 and BioSample Number SAMN44012260.

## References

[B1] Abubakar A. R. Haque M. (2020). Preparation of medicinal plants: basic extraction and fractionation procedures for experimental purposes. J. Pharm. Bioallied Sci. 12 (1), 1–10. 10.4103/jpbs.JPBS_175_19 32801594 PMC7398001

[B2] Antimicrobial Resistance Collaborators (2022). Global burden of bacterial antimicrobial resistance in 2019: a systematic analysis. Lancet 399, 629–655. 10.1016/S0140-6736(21)02724-0 37500506

[B3] Atanasov A. G. Zotchev S. B. Dirsch V. M. Supuran C. T. (2021). Natural products in drug discovery: advances and opportunities. Nat. Rev. Drug Discov. 20, 200–216. 10.1038/s41573-020-00114-z 33510482 PMC7841765

[B4] Bahramian A. Ataie Kachoie M. Rahimi E. (2023). Antimicrobial effects of *Arctium lappa* against infectious bacteria: experimental in vitro analysis. Main. Group Chem. 22, 569–581. 10.3233/MGC-220002

[B5] Balouiri M. Sadiki M. Ibnsouda S. K. (2016). Methods for *in vitro* evaluating antimicrobial activity: a review. J. Pharm. Anal. 6 (2), 71–79. 10.1016/j.jpha.2015.11.005 29403965 PMC5762448

[B6] Bardaji D. K. R. Kumar G. Tran S. Fedus G. Savka M. A. Carter D. (2025a). Whole genome sequencing data of greater burdock (*Arctium lappa*) naturalized in the United States of America. Data Brief. 60, 111508. 10.1016/j.dib.2025.111508 40226200 PMC11986600

[B7] Bardaji D. K. R. Silva N. B. S. Miranda R. R. Martins C. H. G. Hudson A. O. Savka M. A. (2025b). Unlocking the potential of Brazilian plant terpenes to combat antimicrobial resistance. ACS Bio Med. Chem. Au 5, 365–378. 10.1021/acsbiomedchemau.5c00069 40556773 PMC12183609

[B8] Bardaji D. K. R. Kumar G. Tran S. Fedus G. Gan H. M. Savka M. A. (2026). Refining chloroplast genomic resources of *Arctium lappa* through genome skimming and public data mining. Gene Rep. 43, 102499. 10.1016/j.genrep.2026.102499

[B9] Bauer A. W. Kirby W. M. M. Sherris J. C. Turck M. (1966). Antibiotic susceptibility testing by a standardized single disk method. Am. J. Clin. Pathol. 45, 493–496. 10.1093/ajcp/45.4_ts.493 5325707

[B10] Berney M. Hammes F. Bosshard F. Weilenmann H. U. Egli T. (2007). Assessment and interpretation of bacterial viability by using the LIVE/DEAD BacLight kit in combination with flow cytometry. Appl. Environ. Microbiol. 73 (10), 3283–3290. 10.1128/AEM.02750-06 17384309 PMC1907116

[B11] Bolshakova A. V. Kiselyova O. I. Yaminsky I. V. (2004). Microbial surfaces investigated using atomic force microscopy. Biotechnol. Prog. 20, 1615–1622. 10.1021/bp049742c 15575691

[B12] Centers for Disease Control and Prevention (2019). Antibiotic Resistance Threats in the United States, 2019. Atlanta, GA: U.S. Department of Health and Human Services.

[B13] Ciofu O. Tolker-Nielsen T. (2019). Tolerance and resistance of microbial biofilms. Nat. Rev. Microbiol. 17, 621–635. 10.1038/s41579-019-0222-6 35115704

[B14] Clinical and Laboratory Standards Institute (2024). CLSI Standard M100 Performance Standards for Antimicrobial Susceptibility Testing. 34th Ed. Wayne, PA: Clinical and Laboratory Standards Institute.

[B15] Dias D. A. Urban S. Roessner U. (2012). A historical overview of natural products in drug discovery. Metabolites 2, 303–336. 10.3390/metabo2020303 24957513 PMC3901206

[B16] El F. Y. El Barnossi A. Moussaid F. Chahmi N. Al Kamaly O. M. Skali S. N. (2023). Plant-derived natural products as antibiofilm agents against bacterial pathogens. Microorganisms 11, 2123. 10.3390/microorganisms11082123

[B18] Feng X. Tang K. Wang Y. Zhang N. Li S. Luo L. (2025). Assembling the chromosome-level genome of *Arctium lappa* L. provides new insights into carotenoid biosynthesis. BMC Plant Biol. 25, 1508. 10.1186/s12870-025-07539-x 41193967 PMC12590599

[B19] Guedes B. N. Krambeck K. Durazzo A. Lucarini M. Santini A. Oliveira M. B. P. P. (2024). Natural antibiotics against antimicrobial resistance: sources and bioinspired delivery systems. Braz. J. Microbiol. 55, 2753–2766. 10.1007/s42770-024-01410-1 38888693 PMC11405619

[B20] Guimarães A. C. Meireles L. M. Lemos M. F. Guimarães M. C. C. Endringer D. C. Fronza M. (2019). Antibacterial activity of terpenes and terpenoids are present in essential oils. Molecules 24, 2471. 10.3390/molecules24132471 31284397 PMC6651100

[B21] Gupta A. Mumtaz S. Li C. H. Hussain I. Rotello V. M. (2021). Combatting antibiotic-resistant bacteria using nanomaterials. Chem. Soc. Rev. 50, 1881–1934. 10.1039/D0CS01127E PMC6340759 30462112

[B22] Hasan S. A. (2025). Drug resistant bacteria and antimicrobial activity of medicinal plants. Int. J. Nutrol 18 (3). 10.54448/ijn25312

[B23] Huang W. Wang Y. Tian W. Cui X. Tu P. Li J. (2022). Biosynthesis investigations of terpenoid, alkaloid, and flavonoid antimicrobial agents derived from medicinal plants. Antibiotics 11, 1380. 10.3390/antibiotics11101380 36290037 PMC9598646

[B24] Kuete V. (2010). Potential of Cameroonian plants and derived products against microbial infections: a review. Planta Med. 76, 1479–1491. 10.1055/s-0030-1250027 20533165

[B17] Koshak A. E. Elfaky M. A. Abdallah H. M. Albadawi D. A. I. Mohamed G. A. Ibrahim S. R. M. (2024). Arctigenin from burdock root exhibits potent antibacterial and anti-virulence properties against *Pseudomonas aeruginosa* . Microbiol. Spectr. 34 (8), 1642–1652. 10.4014/jmb.2403.03003 39049476 PMC11380511

[B25] Lewis K. Lee R. E. Brötz-Oesterhelt H. Hiller S. Rodnina M. V. Schneider T. (2024). Sophisticated natural products as antibiotics. Nature 632, 39–49. 10.1038/s41586-024-07530-w 39085542 PMC11573432

[B26] Majeed Z. H. Hasan S. A. Ismail R. M. (2022). Evaluate the benefit effects of *Dodonaea viscosa* in kidney of infected rats with *Staphylococcus aureus* . J. Pharm. Negat. Results 13 (3), 291–294. 10.47750/pnr.2022.13.03.046

[B27] Navarro-Muñoz J. C. Selem-Mojica N. Mullowney M. W. Kautsar S. A. Tryon J. H. Parkinson E. I. (2020). A computational framework to explore large-scale biosynthetic diversity. Nat. Chem. Biol. 16, 60–68. 10.1038/s41589-019-0400-9 31768033 PMC6917865

[B28] Newman D. J. Cragg G. M. (2020). Natural products as sources of new drugs over nearly four decades from 01/1981 to 09/2019. J. Nat. Prod. 83, 770–803. 10.1021/acs.jnatprod.9b01285 32162523

[B29] O’Toole G. A. (2011). Microtiter dish biofilm formation assay. J. Vis. Exp. 47, 2437. 10.3791/2437 PMC3182663 21307833

[B30] Parthasarathy A. (2019). Scanning electron microscopy (SEM) for microbes – a simple and inexpensive method for sample preparation. ResearchGate 10.13140/RG.2.2.26222.05449

[B31] Pendleton J. N. Gorman S. P. Gilmore B. F. (2013). Clinical relevance of the ESKAPE pathogens. Expert Rev. Anti Infect. Ther. 11, 297–308. 10.1586/eri.13.12 23458769

[B32] Perez-Delgado O. Alvarado-Pineda R. L. Yacarini-Martinez A. E. (2021). Actividad antibacteriana in vitro de extracto etanólico crudo de las hojas de *Origanum vulgare*, frente a *Staphylococcus aureus* ATCC 29213, *Pseudomonas aeruginosa* ATCC 27853 y *Escherichia coli* ATCC 25922. J. Selva Andina Res. Soc. 12, 21–29. 10.36610/j.jsars.2021.120100021

[B33] Rice L. B. (2008). Federal funding for the study of antimicrobial resistance in nosocomial pathogens: no ESKAPE. J. Infect. Dis. 197, 1079–1081. 10.1086/533452 18419525

[B34] Sarker S. D. Nahar L. Kumarasamy Y. (2007). Microtitre plate-based antibacterial assay incorporating resazurin as an indicator of cell growth, and its application in the in vitro antibacterial screening of phytochemicals. Methods 42, 321–324. 10.1016/j.ymeth.2007.01.006 17560319 PMC1895922

[B35] Tang Y. Lou Z. Rahman M. R. T. Al-Hajj N. Q. Wang H. (2014). Chemical composition and anti-biofilm activity of burdock (*Arctium lappa* L. Asteraceae) leaf fractions against *Staphylococcus aureus* . Trop. J. Pharm. Res. 13, 1933–1939. 10.4314/tjpr.v13i11.23

[B36] Wang H. Joseph J. A. (1999). Quantifying cellular oxidative stress by dichlorofluorescein assay using microplate reader. Free Radic. Biol. Med. 27, 612–616. 10.1016/S0891-5849(99)00107-0 10490282

[B37] Wang D. Bădărau A. S. Swamy M. K. Shaw S. Maggi F. Silva L. E. (2019). *Arctium* species secondary metabolites chemodiversity and bioactivities. Front. Plant Sci. 10, 834. 10.3389/fpls.2019.00834 31338098 PMC6629911

[B38] World Health Organization (2024a). Antimicrobial Resistance. Geneva, Switzerland: World Health Organization.

[B39] World Health Organization (2024b). WHO Bacterial Priority Pathogens List 2024: Bacterial Pathogens of Public Health Importance to Guide Research, Development and Strategies to Prevent and Control Antimicrobial Resistance. Geneva, Switzerland: World Health Organization.

[B40] Yang Y. Li S. Xing Y. Zhang Z. Liu T. Ao W. (2022). The first high-quality chromosomal genome assembly of a medicinal and edible plant *Arctium lappa* . Mol. Ecol. Resour. 22, 1493–1507. 10.1111/1755-0998.13547 34758188

[B41] Yap P. S. X. Yiap B. C. Ping H. C. Lim S. H. E. (2014). Essential oils, a new horizon in combating bacterial antibiotic resistance. Open Microbiol. J. 8, 6–14. 10.2174/1874285801408010006 24627729 PMC3950955

