# Supplementary material for: Characterization of the antibacterial and biofilm inhibitory activities of Arctium lappa L. (greater burdock) against representative members of the ESKAPE group
Source: Front Pharmacol. 2026 Sep 16;17:1941242. doi: 10.3389/fphar.2026.1941242 (PMC13623891; doi:10.3389/fphar.2026.1941242)
Supplement: Supplementary file 1 [file Supplementaryfile1.docx]

| **Solvent** | **Extraction yield (% w/w)** | **Preliminary antibacterial activity*** | **Selected for further study** |
| --- | --- | --- | --- |
| Methanol | 9.57 | + | No |
| Ethanol | 11.65 | + | No |
| Acetone | 5.06 | ± | No |
| Hexane | 2.67 | – | No |
| Ethyl acetate | 5.58 | + | No |
| Aqueous methanol (80:20, v/v) | 9.70 | ++ | No |
| Aqueous ethanol (80:20, v/v) | 10.38 | +++ | Yes |

**Supplementary Table S1**. Extraction yields of *Arctium lappa* leaf extracts obtained with different solvents.

Extraction yield (%) was calculated as the percentage of dried crude extract recovered relative to the initial dry weight of the plant material used for extraction.

**Supplementary Table S2**. GA Best Practice Table 1. Botanical drug and extract characterization

| **Parameter** | **Information** |
| --- | --- |
| Botanical species | *Arctium lappa* L. |
| Plant family | Asteraceae |
| Botanical drug | Leaves |
| Authentication | Plant material was authenticated by whole-genome sequencing and annotation, with additional confirmation by chloroplast genome sequencing and phylogenetic analysis, as previously described by Bardaji et al. (2025a, 2026). |
| Voucher specimen | NCBI BioSample accession SAMN44012260 (BioProject PRJNA1167729). |
| Source | *Arctium lappa* naturalized in a residential garden, Rochester, New York, USA (August 2024). |
| Drying | Freeze-dried. |
| Grinding | Freeze-dried leaves were ground into a fine powder using a laboratory blender. |
| Extraction method | Maceration (1:10, w/v) for 48 h at 20 °C with agitation (80 rpm). |
| Extraction solvents screened | Methanol, ethanol, acetone, hexane, ethyl acetate, 80% aqueous methanol, and 80% aqueous ethanol. |
| Selected extract | 80% aqueous ethanol extract. |
| Drug-to-solvent ratio | 1:10 (w/v). |
| Concentration method | Rotary evaporation under reduced pressure. |
| Storage | Dried extracts stored at 4 °C until use. |
| Extraction yield (%) | Methanol: 9.57%; Ethanol: 11.65%; Acetone: 5.06%; Hexane: 2.67%; Ethyl acetate: 5.58%; 80% aqueous methanol: 9.70%; 80% aqueous ethanol: 10.38%. |
| Phytochemical characterization | Not performed. This study focused on genome-guided prediction and biological evaluation. Metabolomic characterization is planned for future studies. |

**Supplementary Table S3**. GA Best Practice Table 2c. Chemical characterization of the botanical preparation

| **Parameter** | **Information** |
| --- | --- |
| Analytical fingerprint | Not performed. |
| Chromatographic analysis | Not performed. |
| LC–MS | Not performed. |
| GC–MS | Not performed. |
| HPLC fingerprint | Not performed. |
| TLC fingerprint | Not performed. |
| NMR | Not performed. |
| Quantification of marker metabolites | Not performed. |
| Reason | The objective of this study was to investigate the antibacterial activity of a crude 80% aqueous ethanol extract using a genome-guided exploratory approach rather than to chemically characterize the extract. |
| Limitation acknowledged | Yes. The absence of phytochemical characterization and metabolomic validation is explicitly discussed in the revised manuscript. |
| Future work | Bioassay-guided fractionation, metabolomic profiling (LC–MS/MS), and identification of bioactive metabolites are proposed as future studies. |
